# Tragedy and delight: the ethics of decelerated ageing

**DOI:** 10.1098/rstb.2010.0288

**Published:** 2011-01-12

**Authors:** David Gems

**Affiliations:** Institute of Healthy Ageing, and G.E.E., Darwin Building, University College London, Gower Street, London WC1E 6BT, UK

**Keywords:** ageing, decelerated ageing, disease, ethics, longevity

## Abstract

Biogerontology is sometimes viewed as similar to other forms of biomedical research in that it seeks to understand and treat a pathological process. Yet the prospect of treating ageing is extraordinary in terms of the profound changes to the human condition that would result. Recent advances in biogerontology allow a clearer view of the ethical issues and dilemmas that confront humanity with respect to treating ageing. For example, they imply that organismal senescence is a disease process with a broad spectrum of pathological consequences in late life (causing or exascerbating cardiovascular disease, cancer, neurodegenerative disease and many others). Moreover, in laboratory animals, it is possible to decelerate ageing, extend healthy adulthood and reduce the age-incidence of a broad spectrum of ageing-related diseases. This is accompanied by an overall extension of lifespan, sometimes of a large magnitude. Discussions of the ethics of treating ageing sometimes involve hand-wringing about detrimental consequences (e.g. to society) of marked life extension which, arguably, would be a form of enhancement technology. Yet given the great improvements in health that decelerated ageing could provide, it would seem that the only possible ethical course is to pursue it energetically. Thus, decelerated ageing has an element of tragic inevitability: its benefits to health compel us to pursue it, despite the transformation of human society, and even human nature, that this could entail.

## 1. What is biogerontology for?

The last decade has seen many remarkable developments in the biology of ageing, some of them described by other contributors to this special issue. These advances engender not only a sense of excitement and optimism but also, at times, a degree of unease and uncertainty. Questions and comments from members of the public and from journalists present at the recent Royal Society Discussion Meeting on ‘The new science of ageing’ (10–11th May 2010) suggested that some present were wondering, nervously: ‘What do these scientists actually hope to achieve?’ In this essay, I will discuss the goals of biogerontology (the study of the biology of ageing), and their ethical implications.

Much research on the biology of ageing is directed towards one of two goals. The first is intellectual, and one of the greatest remaining challenges to science: an understanding of the ageing process. It is a strange feature of the field of biogerontology that, despite all the recent advances, the fundamental mechanisms of ageing remain uncertain. A dominant idea within the field is that stochastic accumulation of damage to biomolecules is a major driver of ageing, while processes that reduce levels of such damage promote longevity [1]. Yet, this notion has yet to ascend from the status of hypothesis to that of proven fact. The sense of moving at high speed towards the solution of a great mystery makes biogerontology a wonderful field to be working in at the moment.

The second goal of research on ageing is to improve the health of older people. Here, biogerontology is akin to other biomedical research topics, sharing with them the goal of understanding the biological mechanisms that underlie pathology. The particular value of such understanding is that it enables the development of therapeutic treatments, leading to improved health and wellbeing. For example, the identification of bacteria as pathogens eventually leads to the development of antibiotics. But if biogerontology is just another area of biomedical research, does this mean that ageing is a disease, to be treated as if it were the common cold? Should we be seeking a cure for ageing?

## 2. Is ageing a disease?

If something is a disease then one should try to cure it. However, the question of what is a disease and what is not can be a slippery one, and sensitive to cultural perspective. For example, during much of the last century, gay people were viewed as mentally ill. Our view of ageing-related illness is also subject to such nosological elasticity. It was only in 1977 that late-onset Alzheimer's disease (occurring after age 65) was defined as a pathology, as opposed to the natural senility accompanying the second childhood of advanced old age. Is the logical endpoint of this process of redefining isolated components of the ageing process as pathology to view the entire ageing process as pathological?

Whether ageing should be viewed as a disease or a non-pathological process accompanying disease has been debated since classical times [2]. Yet from the perspective of modern biogerontology, there is little to distinguish biological ageing from a disease state [3]. Ageing is a process characterized by a broad spectrum of pathologies the sum of which leads inevitably to death [4], and its biology by loss of homeostasis and accumulation of molecular damage [1]. But is this sufficient grounds to consider it a disease?

Human health has been defined in terms not only of absence of disease, but also in terms of the presence of a level of function that is typical of human beings of a given age and gender [5]. By this view, ageing is a normal process and therefore distinct from disease. Thus, although ageing is characterized by a broad spectrum of diseases, at the same time it is a normal process, and therefore not pathological.

Arguably, this apparent paradox may be resolved by reference to the evolutionary theory of ageing, which provides a biological perspective upon the function and significance of the ageing process [3]. According to the evolutionary theory, ageing is a consequence of a reduction in the force of selection against mutations with deleterious effects later in life [6,7]. This leads to accumulation within populations of alleles with deleterious effects later in life—particularly alleles that also have beneficial effects on reproductive fitness early in life [8]. The evolutionary theory provides the bleak insight that ageing serves no purpose in terms of fitness, but instead is a lethal genetic disease that afflicts all human beings. Arguably, the significance of the universality of ageing is not that ageing is not a disease, but rather that it is a special form of disease. To argue that ageing is not a disease by virtue of its universality is as misleading as it is to argue that the Basenji is not a dog because it does not bark.

The clinical redefinition of ageing as a disease state would not only make sense, but it would also foster the development of therapies of benefit to older people. For example, it would provide incentive to drug companies to profit by developing effective anti-ageing therapies. It would also help to deter those marketing bogus treatments for ageing, and protect older people from being swindled [9,10]. In the USA, for example, the Federal Drug Administration (FDA) is responsible for ensuring the safety and efficacy of medical products at the federal level. Because ageing is not viewed as a disease, orally administered drugs directed against ageing fall under FDA regulations for cosmetic medicine and are, therefore, not subject to same the rigorous testing requirements as other drugs that reduce disease [9,11]. Redefinition of ageing as a disease would lead to proper safety and efficacy testing of anti-ageing treatments.

## 3. How to treat ageing

Recent advances in research on ageing have suggested a number of different approaches that could improve late-life health and wellbeing. Yet, there is no clear consensus on the ideal form that treatments for ageing should take.

### (a) What form should treatments for ageing take?

In terms of treatments for ageing, three different goals of biogerontology may be distinguished, which have been denoted *compressed morbidity*, *arrested ageing* and *decelerated ageing* [11]. With compressed morbidity, the aim is to identify treatments that protect against late-life illnesses without tampering with the underlying ageing process. This would produce improvements in health among the elderly without any major extension of lifespan, and a reduction of the period of disability experienced by elderly people in their final years. This would be beneficial both in terms of improved wellbeing, and lowered healthcare costs. Although wholesome, these aspirations are somewhat unrealistic. For example, they only make sense if ageing and age-related disease are clearly distinguishable, but this is not the case: they are to a high degree part and parcel. Consistent with this, interventions in ageing in laboratory animals have achieved slowed ageing and delayed age-related illness, but not compressed morbidity without life extension. However, the goal of compressed morbidity may, to some degree, be achieved by reducing risk factors for late-life diseases that are distinct from ageing, such as obesity and inactivity.

Arrested ageing involves stopping ageing entirely, or even reversing it, as would be achieved by taking a dip in the mythical fountain of youth. Perhaps, these possibilities will one day be realized, along with others such as interstellar travel, the creation of artificial consciousness and reanimation after cryonic suspension. Yet based on the current state of play in biogerontology, arrested ageing remains a very remote possibility, and is therefore not a topic of any pressing concern.

Unlike the other two goals, decelerated ageing is less an aspiration for what research on ageing should seek to achieve as a view on what it probably can achieve. Work on animal models has shown us that a variety of interventions can slow ageing and, importantly, delay the onset of diseases of ageing [12]. This has important implications in terms of possible future strategies for reducing levels of late-life disease.

### (b) A silver bullet for diseases of ageing?

Much of the serious illness in the developed world is the result of diseases for which ageing is a major risk factor. These include many forms of cancer, cardiovascular disease, neurodegenerative diseases such as Alzheimer's and Parkinson's disease, macular degeneration, arthritis and many, many others. Treating diseases of ageing individually are subject to a law of diminishing returns because of the rapid increase with age in the risk of a broad spectrum of diseases. It is for this reason that successful treatment of individual diseases of ageing yields relatively small increases on life expectancy [13]. For clinicians, the challenge of treating illnesses in the elderly must at times seem like Heracles' trials of combating the multi-headed Hydra. Each time one head was severed, two more would sprout in its place. Likewise, a patient might survive a serious cardiac episode with the help of antihypertensive drugs only to succumb to cancer *and* dementia. Arguably, current research on diseases of ageing fails the Hydra test insofar as it investigates them individually, piecemeal. Researchers studying cancer, cardiovascular disease and neurodegenerative diseases tend to work in separate research institutes, publish in different journals, go to different research conferences and develop treatments that are selectively effective against their target disease.

**Figure 1.**
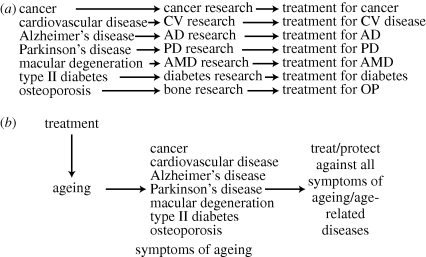
(*a*) A piecemeal approach to study diseases of ageing generates treatments that are limited in scope, and which lead to the rapid replacement of one disease by another (e.g. cardiac ischaemia by Alzheimer's disease). (*b*) A more rational approach to reduce a broad spectrum of age-related diseases would be to treat the underlying cause of all these pathologies: the underlying ageing process. Note that this dichotomous scheme is polemical in character. In fact, recent biomedical studies are increasingly identifying interconnections between late-life pathologies. For example, type 2 diabetes is linked to risk of dementia, driven by vascular disease.

In principle, a more effective way to tackle human age-related illness would be to intervene in ageing itself [14,15]. Deceleration of ageing, demonstrated in animal models, potentially applicable in humans, provides protection against the full spectrum of diseases of ageing thereby assuring late-life health (figure 1), and strikes at the heart of the Hydra of ageing.

## 4. A closer look at decelerated ageing

Current research on the biology of ageing suggests that there is a reasonable possibility that treatments that decelerate human ageing could be developed, given the will to do so. It is therefore worth considering the implications of decelerated ageing more closely.

Clearly, deceleration of ageing could bring great benefits in terms of improved health. But one common concern about treatments for ageing is that they might extend the moribund stage at the end of life. Hospitals might become choked with chronically ill, elderly patients, languishing for years bedbound and miserable, at enormous cost to taxpayers. Animal model studies show that decelerated ageing extends the healthy period of adulthood. However, it could well extend the years of disability too, and result in an expansion of morbidity—at least in absolute terms (i.e. years of life), though perhaps not in relative terms (percentage of life). In this respect, decelerated ageing could prove to be a mixed blessing. Decelerated ageing also has other ethically sensitive implications.

### (a) Decelerated ageing and lifetime risk of disease

**Figure 2.**
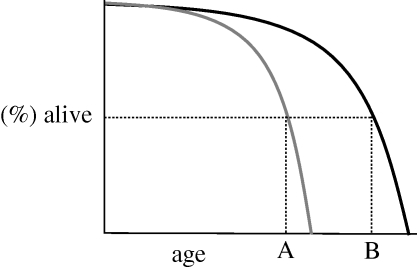
Decelerated ageing may not change lifetime risk of disease. Risk of, say, cancer is greatly reduced at age A in the treated population. However, risk of cancer may well be the same at age A (grey line, untreated) and age B (black line, treated).

A question I am sometimes asked about the effects of decelerated ageing on laboratory animals is: what do they die of? The answer is ageing-related diseases—more specifically, a spectrum of diseases that may or may not differ from those that afflict control, shorter-lived animals. Thus, decelerated ageing is not expected to change the overall, lifetime risk of disease; rather, it reduces disease incidence at any given age. This is well illustrated by the effects of dietary restriction on rodents [16]. Figure 2 depicts schematically the effects of a treatment that decelerates ageing, which extends lifespan and reduces the frequency of ageing-related diseases (e.g. cancer) at any given age, for example age A. However, at age B, where the treated group has reached a state of ageing equivalent to that of the control group at age A, a similar incidence of cancer would be expected. Overall, the lifetime probability of developing ageing-related diseases would be expected to change little.

If lifetime risk of a given disease is unchanged, this might not, at first glance, appear to represent a real improvement in health. However, I believe that such a conclusion would be erroneous, as the following argument illustrates. Consider a disease that is not ageing related: smallpox, caused by the *Variola* virus. The full eradication of this terrible disease in 1977 was one of the greatest achievements of biomedical research. However, one consequence of smallpox eradication was to increase the frequency of late onset Alzheimer's disease (defined as a disease that same year). This it must have done, since it increased the number of individuals surviving to a ripe old age, at which this condition is more frequent.

This illustrates a broader principle: that reduction of one mortal risk often increases exposure to others. The introduction of iron helmets during the First World War led to an increase in the frequency of patients with head injuries by reducing the number of deaths caused by shrapnel. A consequence of the successful control of any given disease is to substitute it for a range of later life pathologies. Since we all die, there is no such thing as lifetime protection against pathology, only deferral of pathology. This deferral is precisely what decelerated ageing would achieve. The only difference is that instead of substituting smallpox now with Alzheimer's later in life it would (in all likelihood) substitute Alzheimer's now for Alzheimer's later. Like curing smallpox now, the latter would be as great an achievement as curing Alzheimer's now.

Yet a lingering doubt remains. Surely smallpox taking the life of a child is more serious than an old woman at the end of her life developing and eventually dying from Alzheimer's disease? Perhaps, but it is an error of youth to think that with age comes a readiness to accept the consequences of ageing, and to tolerate its attendant illnesses. Tolerance of diseases of ageing is grounded in the historically ancient attitude of considering ageing a necessary part of the cycle of life. This tradition, called apologism by the historian Gerald Gruman, is relatively strong in western culture and is, to some extent, rooted in the stoic philosophy of ancient Rome [17].

### (b) The endpoint of decelerated ageing

The example of smallpox can be used to illustrate another odd characteristic of decelerated ageing. One can ask: what should the goals of smallpox research be? Here, the answer is clear: the eradication of smallpox. But consider the consequences of success with respect to decelerated ageing, say using Richard Miller's tentative projection: greatly reduced rates of ageing-related illness, an average lifespan of 112 years, and a maximum lifespan of 140 [18]. Given that lifetime risk of ageing-related illness would not be expected to change, people would still die from diseases of ageing in similar numbers, only later. Thus, the moral imperative driving the research—to relieve suffering from diseases of ageing—would not have been fulfilled; the urgency of further research to decelerate ageing still further would remain. It seems that we would be morally obligated to continue on indefinitely, seeking ever greater decelerations of ageing, ever later postponements of illness and greater extensions of lifespan. Unlike smallpox research, there is no endpoint. This is rather unsettling, and evocative of philosopher Leon Kass's anxious characterization of biomedical research as a ‘runaway train headed to a post-human future’ [19]. Yet, in the end, the shape of the future is so uncertain and subject to future discoveries within the field, and the potential benefits so great, that none of these arguments seem to come even close to being reasons for stopping research that may lead to deceleration of human ageing.

### (c) The dilemma of decelerated ageing

Decelerating human ageing would have two outcomes that are very different in ethical terms. Firstly, it would greatly reduce the frequency of ageing-related illness at any given age. This could achieve an amelioration of human suffering on a very large scale, perhaps comparable in magnitude only to that resulting from the development of antibiotics. This would be a triumph of human endeavour.

Secondly, it would lead to extended lifespan—perhaps, eventually, of a large magnitude. Of course, life expectancy has increased greatly over the course of the last century due to better sanitation and nutrition, and advances in medicine and healthcare provision. This is usually taken as an argument for rather than against Civilization. Yet, the possibility of very large increases in lifespan—let us say, for argument's sake, to 150 years—is one that many find unnerving. Arguably, an intervention resulting in radical life extension would constitute an enhancement technology, belonging in the same category of interventions as cosmetic surgery, cognitive enhancement and perceptual enhancement [20]. Unlike medical treatments, the perceived value of enhancement technologies is often highly sensitive to local differences in cultural perspective, and can involve difficult questions about human identity. Life extension of a large magnitude has these qualities [21]. Thus, decelerated ageing presents a dilemma. Either one must pursue it and reduce suffering but risk extending lifespan to a degree that is socially and existentially problematic; or one must abjure it thereby avoiding the troubles that life extension may cause, but permitting avoidable suffering on a great scale.

Arguably, the only realistic course is the first one. Of these two outcomes of decelerated ageing, protection against disease, and life extension, the first carries far greater moral weight. The possibility of alleviating suffering on such a scale is one that we are morally obligated to pursue, however ambivalent we may feel about the second outcome. Some commentators, including life cycle traditionalists, have argued that ageing is a good thing, such that preventing it to any degree would be wrong [22–24]. But given the health benefits of decelerated ageing, although we may not particularly want life extension (though many, of course, do), we may simply have to accept it as a side effect of a greater benefit. This is a curious circumstance, comparable to imaginary scenarios where a cure for Alzheimer's disease also resulted in cognitive enhancement to far beyond normal capability, or where a new treatment for cardiovascular disease boosted libido to extravagant levels.

Tragic inevitability is part of the human condition and, like theatrical tragedy, one that can possess a strange beauty. The tragic feature of decelerated ageing is the impossibility of separating the aspects of treatment and enhancement. This characteristic effectively compels us to embrace a posthuman future. The only serious option is to adapt as best we can to a future involving ever greater extension of lifespan. With wise and adaptable government in the future, it ought to be possible to achieve this. Yet in the end, the challenge of adjusting to a world where we live much longer, healthy lives is in some respects a delightful one.
